# Surgical treatment of zygomatic bone fracture using two points fixation versus three point fixation-a randomised prospective clinical trial

**DOI:** 10.1186/1745-6215-13-36

**Published:** 2012-04-12

**Authors:** Majeed Rana, Riaz Warraich, Salman Tahir, Asifa Iqbal, Constantin von See, André M Eckardt, Nils-Claudius Gellrich

**Affiliations:** 1 Department of Oral and Maxillofacial Surgery, Hannover Medical School, Hannover, Germany; 2 Department of Oral and Maxillofacial Surgery, King Edward Medical University, Lahore, Pakistan; 3 Department of Craniomaxillofacial Surgery, Hannover Medical School, Carl-Neuberg-Str. 1, 30625 Hannover, Germany

**Keywords:** Zygomatic fracture, Open reduction, Internal fixation, Three point fixation, Two point fixation

## Abstract

**Background:**

The zygoma plays an important role in the facial contour for both cosmetic and functional reasons; therefore zygomatic bone injuries should be properly diagnosed and adequately treated. Comparison of various surgical approaches and their complications can only be done objectively using outcome measurements which in turn require protocol management and long-term follow up. The preference for open reduction and internal fixation of zygomatic fractures at three points has continued to grow in response to observations of inadequate results from two point and one point fixation techniques.

The objectives of this study were to compare the efficacy of zygomatic bone after treatment with ORIF using 2 point fixation and ORIF using 3 point fixation and compare the outcome of two procedures.

**Methods:**

100 patients were randomly divided equally into two groups. In group A, 50 patients were treated by ORIF using two point fixation by miniplates and in group B, 50 patients were treated by ORIF using three point fixation by miniplates. They were evaluated for their complications during and after surgery with their advantages and disadvantages and the difference between the two groups was observed.

**Results:**

A total of 100 fractures were sustained. We found that postoperative complication like decreased malar height and vertical dystopia was more common in those patients who were treated by two point fixation than those who were treated with three point fixation.

**Conclusions:**

Based on this study open reduction and internal fixation using three point fixation by miniplates is the best available method for the treatment zygomatic bone fractures.

## Background

The face occupies the most prominent position in the human body rendering it vulnerable to injuries quite commonly. The prominence of the zygomatic region predisposes it to bearing the brunt of the facial injuries [1]. Because of its position, it is the second most common mid-facial bone fractured after the nasal bones and overall represents 13% of all craniofacial fractures [1,2].

However, the incidence and etiology varies from area to area as another study shows that zygomatic bone fractures were commonly found among young males and the most common cause was found to be road traffic accidents [3].

The sex distribution is markedly higher for males than for females (4:1). In developed countries, the ratio is on average 3-5:1, whereas in underdeveloped countries, the ratio is on average 10-40:1 [2].

The causes of the fractures were mainly attributed to assault and road traffic accidents (RTA), which is iconsistent with worldwide experience. However, in many places, either RTA or assault was consistently the main contributing cause with one of these two consistently dominating the other by a large degree [2].

The architectural pattern of zygomatic bone allows it to withstand blows of great forces without fracturing. Because of such heavy forces zygomatic bone gets separated from adjacent bone at or near the suture lines. It may be separated from its four articulations, resulting in a zygomatico-maxillary complex, zygomatic-complex or orbito-zygomatic fracture. Fractures of this complex are one of the more common types of maxillofacial injuries to treat. They are seen as isolated or in association with other facial fractures due to the complex midface anatomy [4-6].

The fracture of the zygomatic bone can result in restricted mouth opening due to impingement on the coronoid process. Disruption of the zygomatic position also carries psychological, aesthetic and functional significance, causing impairment of ocular and mandibular function. Therefore, for both cosmetic and functional reasons, it is mandatory that zygomatic bone injury is properly diagnosed and adequately managed [7].

Skeletal healing of displaced zygomatic bone fragments after insufficient fracture reduction and fixation results in an inadequate projection of the zygomatic body and thus facial asymmetry. Accurate assessment of the position of the zygomatic bone in relation to the cranial base posteriorly and the midface anteriorly, is the key to the acute repair of mid facial fractures. Secondary reconstruction of posttraumatic deformities of the orbitozygomatico- maxillary complex remains a major surgical challenge.

Three principle buttresses need to be considered in midface fractures. The medial or nasomaxillary buttress reaches from the anterior maxillary alveolus to the frontal cranial attachment. The second is the pterygomaxillary or posterior buttress, which connects the maxilla posteriorly to the sphenoid bone. The third is the lateral or zygomaticomaxillary buttress. This important buttress connects the lateral maxillary alveolus to the zygomatic process of the temporal bone. These buttresses help to give the zygoma an intrinsic strength such that blows to the cheek usually result in fractures of the zygomatic complex at the suture lines, rarely of the zygomatic bone itself [8].

Another important landmark with respect to zygomatic fractures is the sphenozygomatic junction (especially laterally displaced fractures). The alignment of the zygoma with the greater wing of the sphenoid in the lateral orbit is critical for determining adequate reduction of zygomatic fractures. Reducing the three points that make up the buttresses also helps to ensure proper alignment of the zygoma and proper reduction of other facial fractures present. This graduated approach helps to preserve facial height and width [8].

Various surgical techniques have been described for the reduction of zygomatic complex fracture. Open reduction with surgical incisions has been accomplished through Keen's approach, Gillies' approach, bicoronal scalp flap approach or the more popular Dingman's approach. Gillies' approach is the temporal approach. This procedure has advantages in that it leaves no facial scars and is simple to perform. The Gillies temporal approach method is used widely in U.K for zygomatic bone fracture [9,10].

Open reduction & internal fixation of simple displaced fractures of the zygoma in an attempt to define the simplest method of achieving post reduction stability. In a report, the three-point fixation (FZ suture, inferior orbital rim, and zygomaticomaxillary buttress) using either miniplates alone or interfragmentary wiring conferred the greatest stability [11].

Comparison of various surgical approaches and their complications can only be done objectively using outcome measurements which in turn require protocol management and long-term follow up. The preference for open reduction and internal fixation of zygomatic fractures using three point fixation has continued to grow in response to observations of inadequate results from two pint fixation technique, with the exception of management of isolated fractures of the zygomatic arch [12].

Miniplate removal following trauma surgery is indicated in approximately 10% of cases and is mainly caused by infection and/or dehiscence, pain, interference with denture position, screw or plate failure, and palpability [13].

This study was designed to compare 2 point internal fixation with 3 point internal fixation, for the better clinical results and fewer complications, consequently contributing towards the greater goals of a better treatment option and in due process benefit the concerned patients.

## Methods

The study was approved by the local ethics committee at the King Edward Medical University Lahore (F-07-2932). Trial was approved and registered at the research, training and monitoring cell, College of Physicians & Surgeons, Pakistan. RTMC allotted registered number: DSG-2007-066-486. Before the beginning of the study, written informed consent was obtained from each patient.

### Patients

100 healthy patients were scheduled for treatment of zygomatic bone fractures. Only patients who required open reduction and internal fixation were divided randomly into 2 treatment groups. 50 patients were treated with open reduction and internal fixation using 2 point fixation technique and the other 50 patients were treated with open reduction and internal fixation using 3 point fixation technique. Intraoral Keens approach and Gillies temporal approach were used for reducing the fractures. The points of fixation were frontozygomatic suture, zygomatico maxillary buttress region and infraorbital margin. Patients with 2 point fixation technique had fixation done at frontozygomatic suture and zygomatico maxillary buttress region while the patients with 3 point fixation technique had fixation done at frontozygomatic suture, infraorbital margin and zygomatico maxillary buttress region. The observer did not know about the kind of therapy applied at the time of the patient examinations. Surgeons treating the patients were blinded to the randomization scheme. The patients were not blinded because they were informed that the study was designed to compare the 2 point fixation technique with 3 point fixation technique on malar height and vertical dystopia.

### Consort flow diagram

At the time of presentation 166 patients were assessed for eligibility to be included in the study. Out of these 25.3% of the patients (n = 42) were not included in the study as 18.67% patients (n = 31) did not meet the inclusion criteria while 6.67% (n = 11) did not want to participate in the study. A total of 124 patients were randomly allocated in two groups with 62 patients allocated in each group for intervention. In the two point fixation group 96.7% patients (n = 60) received the selected intervention while 3.23% patients (n = 2) patients have to be excluded as the patients could not afford miniplates and screws. In the three point fixation group 95.1% patients (n = 59) received the selected intervention while 4.9% patients (n = 3) patients have to be excluded as the patients could not afford miniplates and screws. Among the 60 patients who received intervention in 2 point fixation group 13.3% (n = 8) were lost to follow-up as these patients come from far areas and could not travel due to economic or personal reasons. While the 59 patients who received intervention in 3 point fixation group 10.2% (n = 6) were lost to follow-up. The 52 patients who received the treatment in 2 point fixation group and were available for follow-up, 2 of them had their data lost during the data analysis procedure. So the total number of patients who were analyzed in 2 point fixation were 50.

The 53 patients who received the treatment in 3 point fixation group and were available for follow-up, 3 of them had their data lost during the data analysis procedure. So the total number of patients who were analyzed in 2 point fixation were 50.

### Randomisation

Randomisation was done using a computer based software "EpiCalc2000". The software was used to generate serial numbers 1-100 into two groups randomly and those patients who fulfilled the inclusion criteria were allocated serial numbers according to date and sequence of admission to hospital. The person responsible for conducting the measurements at the time of assessment of variables was blindfolded regarding the type of procedure that was conducted.

### Reduction methods

Gillies Temporal approach uses a 2.5 cm incision, inclined at an angle of 45° to the zygomatic arch, in the temporal region in the hear bearing area of scalp. The Rowe zygoma elevator is inserted between the fascia and Temporalis muscle and fracture is reduced. Keens approach uses a small incision of approximately 1 cm made in the mucobuccal fold just beneath the zygomatic buttress of the maxilla. Elevator is passed upwards behind the fractured bone maintaining close contact with the bone in order to avoid entering the fat pad in the temporal area. Reduction is achieved by elevating the bone upward and outward: a snapping sound may be heard when the bone is replaced

### Fixation methods

The approach to expose the fracture sites was achieved using different standard incisions. In patients with 2 point fixation technique the frontozygomatic suture was approached using a lateral eyebrow incision or a upper lid blephroplasty incision. The zygomatico maxillary buttress was exposed using intraoral buccal sulcus incision. In patients with 3 point fixation additional exposure of infraorbital rim was accomplished using subciliary incision or a transconjunctival approach.

The fixation method sued was 1.5 mm miniplates at frontozygomatic suture and zygomatico maxillary buttress region while 0.9 mm microplates were used to fix the infraorbital margin. Patients with 2 point fixation technique had fixation done at frontozygomatic suture and zygomatico maxillary buttress region while the patients with 3 point fixation technique had fixation done at frontozygomatic suture, infraorbital margin and zygomatico maxillary buttress region

### Study including criteria's and protocol

The study included patients aged between 14 and 60 years with isolated zygomatic bone fractures. The study included laterally displaced Zygomatic bone fracture as determined on clinical and radiographic findings (Waters' view, Caldwell's posterior-anterior view) and patients with zygomatic bone fracture displaced in other directions but more than 15 days old. Patients who had gun shot injuries and communited fractures of zygomatic bone or patients who were medically unfit for surgery or to undergo general anesthesia were excluded from the study. The clinical inclusion and exclusion criteria's are shown in Table 1. Preoperatively all patients were thoroughly examined and investigated using Waters' view, Caldwell's posterior-anterior view. Preoperatively malar height was measured from vertex view of the patient comparing fractured site with normal site and measuring with a vernier calliper. For measurement of malar height a single reference point (intersection point of midsagittal line with the intercanthal line) was taken and second point was taken at the maximum height of malar region as viewed from vertex view of patient and distance was measured between these two points preoperatively and post operatively.

**Table 1:** Study inclusion and exclusion criteria

Inclusion criteria	Exclusion criteria
Patients with isolated, laterally displaced Zygomatic bone fracture determined on clinical and radiographic findings (Waters' view, Caldwell's posterior-anterior view). Patients with zygomatic bone fracture displaced in other directions but more than 15 days old. Intra-oral approach	Comminuted zygomatic bone fracture Gun shot injuries Medically unfit for surgery, who are unfit to undergo General Anesthesia as evident from pre operative anesthetic evaluation Infected fractures Pathological fractures Open fracture extra-oral approach

Age between 14 and 60	missing operability foreseeable missing opportunity of follow-up examination

Written informed consent	pregnancy, heart-, pulmonal-, liver- and kidney disease, chronic pain syndrom nursing, drug addiction, recent operations, and diseases like heart, metabolism, CNS, infectious, circulation, systemic, malignant and immune system affecting diseases as well as blood coagulation disorders and allergic reactions to pharmaceuticals and antibiotics

Vertical dystopia was measured preoperatively and postoperatively as difference in level of bony orbits indicated by palpation and comparing with normal side measured by a scale on Waters view using a tracing paper to outline the infraorbital margin.

### Post-operative malar height analysis

Post-operative malar height analysis was conducted with the method already described above at 1 st, 3rd and 6th week after operation, where the patients malar height was recorded in a proforma. At the sixth week Malar height will be confirmed, completing the six weeks follow up assessment.

### Post-operative vertical dystopia analysis

Post-operative vertical dystopia analysis was conducted with the method already described above at 1st, 3rd and 6th week after operation, where the patients vertical dystopia was recorded in a proforma. At the sixth week study, vertical dystopia will be confirmed, completing the six weeks follow up assessment.

### Statistical analysis

Data was analyzed by SPSS version 14.0, a computer based software. Quantitative variable, age, Malar height, vertical dystopia has been presented as Mean ± SD. t-Test was used for comparison between the two groups. P ≤ 0.05 was taken as significant except age.

## Results

**Figure 1:**
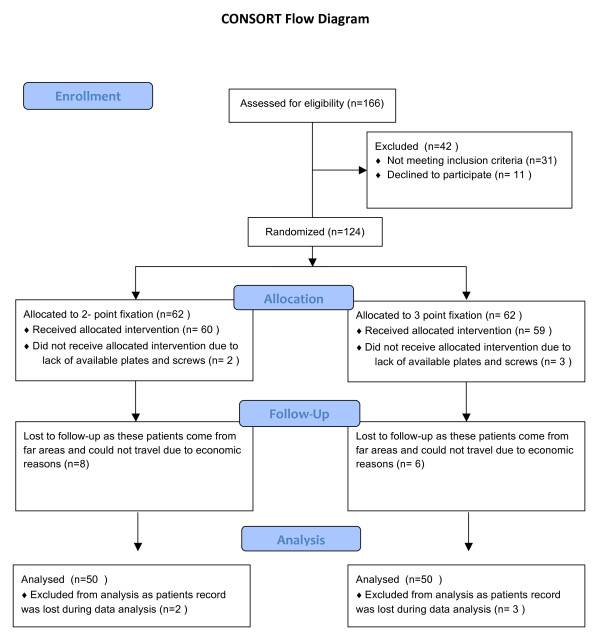
**Demonstrates the consort flow diagram**.

In this study 100 patients were randomly divided into two study groups. In Group A, patients were treated with 2 point fixation and in Group B the patients were treated with 3 point fixation. The clinical and demographic characteristics of patients in both groups are shown in Table 2, Figure 1.

**Table 2:** Baseline characteristics of patients

	2 point fixation	3 point fixation	*P *value
Gender female--no./total no. (%)	6/50 (12)	9/50 (18)	0.67
Age (years) ± SD	31.60 ± 12.35	30.34 ± 11.7	0.601
Operation duration (minutes) ± SD	97.8 ± 52.7	99.5 ± 31.2	0.92
Hospitalization duration (days) ± SD	6.2 ± 1.2	6.1 ± 1.5	0.812

### Age distribution

The average age of patients in Group A was 31.60 ± 12.35 years with age range 51 (68-17) years while in Group B the average age was 30.34 ± 11.69 years with age range 55 years (60-15) years. In both study groups, there was no statistical difference in the average age of the patients, i.e. p-value (0.601 > 0.05). Table 3

**Table 3:** Descriptive Statistics of Age (in years)

Age (in years)		Study Groups		
		
		A	B	Total
	N	50	50	100
	
	Mean	31.60	30.34	30.97
	
	Std. Deviation	12.35	11.69	11.98
	
	Std. Error	1.74	1.653	1.198
	
	Minimum	17	15	15
	
	Maximum	68	60	68

Study Group A = 2 point fixation

Study Group B = 3 point fixation

t- test = 0.524

p-value = 0.601

### Gender distribution

According to the gender there were 85 male patients in which 44 were treated with 2 point fixation while the rest of 41 male were treated with 3 point fixation. There were only 15 female patients in this study, in which 6 were treated with 2 point fixation and 9 were treated with 3 point fixation. The male to female ratio was 5.67: 1 in this study.

### Malar height

In group A, the average malar height at 1st week was 69.18 ± 3.19 mm with range 14 (75-61) mm. In group B, the average malar height at 1st week was 69.02 ± 3.25 mm with range of 14 (75-14) mm. The difference in malar height was statistically insignificant in both study groups i.e. p-value (0.801 > 0.05). Table 4

**Table 4:** Descriptive Statistics of Malar height (mm) at 1st week

Outcome at 1st Week for Malar Height (mm)		Study Groups		
		
		A	B	Total
	N	50	50	100
	
	Mean	69.18	69.02	69.10
	
	Std. Deviation	3.192	3.25	3.21
	
	Std. Error	0.45	0.46	0.32
	
	Minimum	61.00	61.00	61.00
	
	Maximum	75.00	75.00	75.00

Study Group A = 2 point fixation

Study Group B = 3 point fixation

t- test = 0.248

p-value = 0.804

At third week, the average malar height in group A was 67.02 ± 3.52 mm with range 16 (75-59) mm, while in group B the average malar height was 68.38 ± 3.62 with range of 13 (74-61) mm. At third week the average malar height was statistically same (insignificant) i.e. p-value (0.06 > 0.05). Table 5

**Table 5:** Descriptive Statistics of Malar height (mm) at 3rd week

Outcome at 3^rd ^Week for Malar Height (mm)		Study Groups		
	
		A	B	Total
	N	50	50	100
	
	Mean	67.02	68.38	67.70
	
	Std. Deviation	3.52	3.62	3.62
	
	Std. Error	0.50	0.51	0.36
	
	Minimum	59.00	61.00	59.00
	
	Maximum	75.00	74.00	75.00

Study Group A = 2 point fixation

Study Group B = 3 point fixation

t- test = -1.90

p-value = 0.060

The final outcome of malar height was measured at 6th weeks so; the average malar height in group A was 66.72 ± 3.62 mm with minimum and maximum value 59 mm and 75 mm respectively. In group B the average malar height at 6th week was 68.26 ± 3.76 mm with minimum and maximum value 60 mm and 74 mm respectively. According the measurement of malar at 6th week, the malar height was statistically significant (i.e. the average malar is greater in group B) i.e. p-value (0.04 < 0.05). Table 6

**Table 6:** Descriptive Statistics of Malar height (mm) at 6th week

Outcome at 6^th ^Week for Malar Height (mm)		Study Groups		
	
		A	B	Total
	N	50	50	100
	
	Mean	66.72	68.26	67.49
	
	Std. Deviation	3.62	3.76	3.75
	
	Std. Error	0.51	0.53	0.53
	
	Minimum	59	60.00	59
	
	Maximum	75	74.00	75

Study Group A = 2 point fixation

Study Group B = 3 point fixation

t- test = -2.086

p-value = 0.04

### Vertical dystopia

Moreover, at first week the vertical dystopia of group A was 1.84 ± 0.68 mm with range of 2 (3-1) mm and in group B the average vertical dystopia was 1.86 ± 0.77 with range of 3 (3-0) mm. The average vertical dystopia at 1st week was statistically insignificant i.e. p-value (0.897 > 0.05). Table 7

**Table 7:** Descriptive Statistics of Vertical Dystopia at 1st week

Outcome at 1st Week for Vertical Dystopia (mm)		Study Groups		
	
		A	B	Total
	N	50	50	100
	
	Mean	1.84	1.86	1.85
	
	Std. Deviation	0.68	0.86	0.77
	
	Std. Error	0.096	0.12	0.077
	
	Minimum	1.00	.00	.00
	
	Maximum	3.00	3.00	3.00

Study Group A = 2 point fixation

Study Group B = 3 point fixation

t- test = -0.129

p-value = 0.897

In group A, at 3 rd week the average vertical dystopia was 2.96 ± 0.924 mm with minimum and maximum value 1 mm and 5 mm respectively. Similarly the average vertical dystopia in group B was 2.28 ± 1.05 mm. The minimum and maximum value of vertical dystopia was 0 mm and 5 mm respectively. In group A, the average vertical dystopia at 3rd week was statistically greater i.e. p-value (0.001 < 0.05, significant). Table 8

**Table 8:** Descriptive Statistics of Vertical Dystopia at 3rd week

Outcome at 3^rd ^Week for Vertical Dystopia (mm)		Study Groups		
	
		A	B	Total
	N	50	50	100
	
	Mean	2.96	2.28	2.62
	
	Std. Deviation	0.92	1.050	1.04
	
	Std. Error	0.13	0.148	0.104
	
	Minimum	1.00	.00	.00
	
	Maximum	5.00	5.00	5.00

Study Group A = 2 point fixation

Study Group B = 3 point fixation

t- test = 3.435

p-value = 0.001

Finally, in group A, at 6th week the average vertical dystopia was 3.18 ± 1.003 mm with range 4 mm (5-1) mm and in group B the average vertical dystopia was 2.36 ± 1.102 mm with range 3 mm (3-0) mm. The average vertical dystopia was higher in group A as compared to group B. Hence the average vertical dystopia was statistically significant at 6th week i.e. p-value (0.000 < 0.05). Table 9

**Table 9:** Descriptive Statistics of Vertical Dystopia at 6th week

Outcome at 6^th ^Week for Vertical Dystopia (mm)		Study Groups		
	
		A	B	Total
	N	50	50	100
	
	Mean	3.18	2.36	2.77
	
	Std. Deviation	1.003	1.102	1.126
	
	Std. Error	0.14	0.155	0.112
	
	Minimum	1.00	0	0
	
	Maximum	5.00	3.00	5.00

Study Group A = 2 point fixation

Study Group B = 3 point fixation

t- test = 0.381

p-value = 0.000

According to the final assessment, fractures stability was seen in 56 patients in which 16 patients were from group A and 40 patients were from group B. Thirty-four fracture were unstable in group A and 10 were unstable in group B. The fracture stability was statistically higher in Group B as compared to group A, i.e. p-value (0.000 < 0.05). Table 10, Table 11.

**Table 10:** Final Assessment

		Final Assessment		Total
			
		Stable	Unstable	
**Study Groups**	A	16	34	50
	
	B	40	10	50

Total		56	44	100

Study Group A = 2 point fixation

Study Group B = 3 point fixation

Chi-squares = 23.37

p-value = 0.000

**Table 11:** Final Results and P value

	2 point fixation	3 point fixation	P value
Malar Height 1^st ^week(mm) ± SD	69.18 ± 3.2	69.02 ± 3.25	0.804
Malar Height 3^rd ^week(mm) ± SD	67.02 ± 3.52	68.38 ± 3.62	0.060
Malar Height 6^th ^week(mm) ± SD	66.72 ± 3.62	68.26 ± 3.76	0.04
Vertical Dystopia 1^st ^week(mm) ± SD	1.84 ± 0.68	1.86 ± 0.86	0.897
Vertical Dystopia 3^rd ^week(mm) ± SD	2.96 ± 0.92	2.28 ± 1.05	0.001
Vertical Dystopia 6^th ^week(mm) ± SD	3.18 ± 1.003	2.36 ± 1.102	0.000
Final Assessment -stable/unstable (stable%)	16/34(32)	40/10(80)	0.000

## Discussion

Most of the studies about the Zygomatic bone fracture have not been designed adequately to provide meaningful comparison. Displaced zygoma fractures are vulnerable to secondary malposition as a result of masticatory forces even after some kind of fixation [14]. These forces must be overcome at fracture sites for optimal stabilization [15]. Any post-reduction displacement of zygoma can result in delayed development of malar asymmetry and vertical dystopia. Therefore the goal of treatment of zygomatic fractures is to restore and maintain pre-injury facial skeletal configuration. The biomechanics of the facial skeleton were investigated and discussed by Rudderman and Mullen [16]. According to them, fractured zygomatic segment has six possible directions of motion: translation across x, y and z axis; rotation about x, y and z axis. A miniplate applied across the fronto-zygomatic suture will resist translatory movement and also rotation along an axis perpendicular to the plane of miniplate because of the width of the plate. At the same time, it will offer little resistance to rotation along the linear axis of the plate. To improve stabilization, an additional plate is to be applied in a manner where the weak axis of both plates does not coincide with a line connecting them. A still more favorable situation can be created by choosing three fixation points that are not collinear. According to Pearl [17], it is essential to reposition the zygoma at a minimum of three locations to achieve correction in three dimensions. He further opined that reduction at the fronto-zygomatic suture and inferior orbital rim can still leave persistent lateral rotation in the region of the anterior maxillary buttress leading to intra-orbital volume expansion behind the axis of globe. Many experimental biophysical studies have been conducted to find out post-reduction rotational stability of zygoma fracture after miniplate fixation. Davidson et al [18] analyzed different combinations of miniplate fixation for stabilizing fractured zygoma in human skulls. This experimental study found that three-point fixation at fronto-zygomatic suture; inferior orbital rim and zygomatico-maxillary buttress conferred maximum stability against forces matching physiological stresses. Similar results were found by O'Hara et al [19] in another experimental biophysical study. Despite these experimental studies, there were no prospective clinical studies.

The difference studies that were conducted to show that one point fixation [20] and two point fixation [21] also show good results, were primarily aimed to reduce the scar mark of incision. If the incisions are properly made using the option of transconjunctival incision for orbital rim (which leaves no obvious scar), upper eyebrow incision for FZ suture (minimal scar that can hide under eyebrow) and intraoral buccal sulcus incision (no visible scar), the 3 point fixation can give us better esthetics results.

Despite these apparent advantages, three-point fixation is associated with more extensive periosteal stripping, extreme retraction of bone edges and requirement of expert assistance for application of miniplate across the zygomatico-maxillary buttress. In addition, longer operative time, presence of more hardware and increase in cost of surgery are some disadvantages of fixation across an additional point. However, in light of the literature review, it was found out that irrespective of the approach taken for reduction, good results can be achieved by ensuring that the zygomatic bone fractures are properly reduced and adequately stabilized at atleast three points. Concerning the treatment of the Zygomatic bone fracture, however we have tried to provide some valuable information about the two different treatment options. The mean age group in my study was 30 to 31 (group A, mean 31.60 and Group B mean age 30.34) years. Third decade constituted the major group in this study, which is the same as previous studies by Haider Z (1977) [21], Adekey (1980)[22], Shepherd (1990)[23], Tanaka (1994)[24], and Anwar et al (1998)[25], Fasola AO (2002)[26].

The adult is more vulnerable due to dominant outdoor activities at that stage of life, specially fights and high speed transportation as reported by Adekeye (1980)[22].

It appears from our data that majority of our patients presenting with Zygomatic bone fractures were males and a considerably lower proportion of patients were females. The male to female ratio was 5.67: 1. Most other studies similarly indicate a male prediction with ratio of approximately 4:1 to female [27]. In developed countries, the ratio is on average 3-5: 1 [28] whereas in underdeveloped countries, the ratio is on average 10-40:1 [22,29]. Our ratio compares favorably with that of Ugboko V et al ^90 ^(2005) with ratio as 6:1 and Fasola [26] (2002) with ratio 5.4:1.

The sex distribution is markedly higher for males than for females in our society because females are more confined to indoor activities whereas males are more exposed to external environment during commuting as well as during their jobs. The approach to zygomatic bone fracture was directed to the FZ suture, infraorbital rim and zygomatico maxillary buttress. The Fronto Zygomatic suture can be exposed using standard upper eyebrow incision. The inferior orbital rim can be exposed via an infraciliary, infraorbital or transconjunctival approach. The transconjunctival incision gives excellent exposure and saves the patient a visible scar on the face. Typically, the inferior rim defects are visible through the orbital incision. The lateral rim fracture frequently occurs at the FZ suture line. This sometimes can be reached via the lateral lid crease or canthal incision. Rarely, a second incision may be needed under the lateral brow. This can be used to approach the lateral fracture and provide access to elevate that bony fragment. Once proper access to the lateral rim has been achieved, an elevator is passed along the lateral rim and under the zygomatic arch at its anterior origin. Firm anterior pressure, not prying, is applied to the elevator to align the lateral and inferior fragments. Once these are positioned, they are fixated with miniplates. The zygomatico maxillary buttress can be approached intraorally using the buccal sulcus incision. Fracture segments can be directly visualized, reduced and fixed using this approach. Precise reconstruction with rigid internal fixation of the zygoma at 3 points (across the frontozygomatic suture, the inferior orbital rim, and the lateral midfacial buttress) is needed to counter the force of the masseter muscle.

## Conclusions

Fracture of zygomatic bone is more common in adult males who are more exposed to external environment as compared to females. Assessment of objective post fixation variables, i.e. vertical dystopia, and malar height show statistically significant enhancement in outcome attesting to better inherent stability of three-point fixation. Considering zygomatic bone fracture as a tetrapod fracture we recommend that for laterally displaced and unstable fractures rigid internal fixation should be done at atleast three points using miniplates.

### Clinical relevance

This study provides modern treatment strategies for treatment of zygomatic fracture.

## Competing interests

The authors declare that they have no competing interests.

## Authors' contributions

MR, RW, ST, AI, CS, AME and NCG conceived of the study and participated in its design and coordination. MR and ST made substantial contributions to data acquisation and conception of manuscript. MR and ST drafted and designed the manuscript. MR performed the statistical analysis. NCG, RW and AME were involved in revising the manuscript. All authors read and approved the final manuscript.

## Consent statement

Written informed consent was obtained from the patient for publication of this research and accompanying images. A copy of the written consent is available for review by the Editor-in-Chief of this journal.

## Funding

The article processing charges are funded by the Deutsche Forschungsgemeinschaft (DFG), "Open Acess Publizieren".

## References

[B1] Chowdhury SKR Menon PS Etiology and management of zygomatico-maxillary complex fractures in the armed forces MJAFI 2005 61 238 240 10.1016/S0377-1237(05)80162-5 PMC4925444 27407768

[B2] Tadj A Kimble FW Fractured zygoma ANZ J Surg 2003 73 49 54 10.1046/j.1445-2197.2003.02595.x 12534741

[B3] Cheema SA Zygomatic bone fracture J Coll Physicians Surg Pak 2004 12 815 821 15233885

[B4] Ho V Isolated bilateral fractures of zygomatic arches Br J Oral Maxillofac Surg 1994 32 394 10.1016/0266-4356(94)90033-7 7849002

[B5] Medvedev IA Sivolapov KA The use of titanium devices in treating fractures of the zygomatico-orbital complex Stomatologiia (Mosk) 1993 72 1 19 23 8048091

[B6] Crowe WW Treatment of depressed fracture of the zygomatic bone J Oral Surg 1952 10 3 14898342

[B7] Nayyar MS Management of zygomatic complex fracture J Coll Physicians Surg Pak 2002 12 700 705

[B8] Rohrich RJ Watumull D Comparison of rigid plate versus wire fixation in the management of zygoma fractures: a long-term follow-up clinical study Plast Reconstr Surg 1995 96 3 570 575 10.1097/00006534-199509000-00008 7638281

[B9] Lee PK Lee JH Choi YS Single transconjunctival incision and two-point fixation for the treatment of noncomminuted zygomatic complex fracture J Korean Med Sci 2006 21 1080 1085 10.3346/jkms.2006.21.6.1080 17179691 PMC2721933

[B10] Courtney DJ Upper buccal sulcus approach to management of fractures of the zygomatic complex: a retrospective study of 59 cases Br J Oral Maxillofac Surg 1999 37 464 468 10.1054/bjom.1999.0010 10687908

[B11] Davidson J Nickerson D Nickerson B Zygomatic fractures: Comparison of method of internal fixation Plast Reconstr Surg 1990 86 25 32 10.1097/00006534-199007000-00004 2359799

[B12] David DJ Facial fracture classification: current thoughts and applications J Craniomaxillofac Trauma 1999 5 31 36 11951263

[B13] Mosbah M Oloyede D Koppel D Moos K Stenhouse D Miniplate removal in trauma and orthognathic surgery--a retrospective study Int J Oral Maxillofac Surg 2003 32 148 151 10.1054/ijom.2002.0344 12729774

[B14] Manson PN Crawley WA Yaremchuk MJ Midface fractures: Advantages of immediate extended open reduction and bone grafting Plast Reconstr Surg 1985 76 1 9 10.1097/00006534-198507000-00001 3892561

[B15] Rohrich RJ Hollier LH Watumull D Optimizing the management of orbitozygomatic fractures Clin Plast Surg 1992 19 149 165 1537216

[B16] Rudderman RH Mullen RL Biomechanics of facial skeleton Clin Plast Surg 1992 19 11 29 1537212

[B17] Pearl RM Treatment of enophthalmos Clin Plast Surg 1992 19 99 111 1537231

[B18] Davidson J Nickerson D Nickerson B Zygomatic fractures: comparison of methods of internal fixation Plast Reconstr Surg 1990 86 25 32 10.1097/00006534-199007000-00004 2359799

[B19] O'Hara DE Delvecchio DA Bartlett SP The role of microfixation in malar fractures: a quantitative biophysical study Plast Reconstr Surg 1996 97 345 353 10.1097/00006534-199602000-00011 8559817

[B20] Fujioka M Yamanoto T Miyazato O Nishimura G Stability of one-plate fixation for zygomatic bone fracture Plast Reconstr Surg 2002 109 817 818 10.1097/00006534-200202000-00068 11818879

[B21] Haider Z Fractures of the zygomatic complex in South East Region of Scotland Br J Oral Surg 1978 15 265 267 10.1016/0007-117X(78)90011-2 272925

[B22] Adekeye EO Fracture of zygomatic complex in Nigerian patient J Oral Surg 1980 38 596 599 6930461

[B23] Shepherd JP Shapland M Scullye S Leslie IJ Alcohol intoxication and severity of injury in assault Br Med J 1988 296 1299 1303 10.1136/bmj.296.6632.1299 3133057 PMC2545769

[B24] Tanaka ON Tomitsuka K Shionoya K Andou H Kiimijima Y Etiology of maxillofacial fractures Br J Oral Maxillofac Surg 1994 32 19 23 10.1016/0266-4356(94)90166-X 8136332

[B25] Anwar BB Etiology and incidence of maxillofacial fractures in north of Jordan Oral Surg Oral Med Oral Pathol 1998 86 31 35 10.1016/s1079-2104(98)90146-9 9690242

[B26] Fasola AO Obiechina AE Arotiba JT Zygomatic complex fractures at the University College Hospital, Ibadan, Nigeria East Afr Med J 2002 79 11 13 12389959 10.4314/eamj.v79i3.8892

[B27] Marciani RD Caldwell GT Hall J Maxillofacial injuries associated with all terrain vehicles J Oral Maxillofac Surg 1999 57 119 123 10.1016/S0278-2391(99)90221-5 9973117

[B28] Covington DS Wainwright DJ Teichgraeber JF Parks DH Changing patterns in the epidemiology and treatment of zygoma fractures: 10-year review J Trauma 1994 37 243 248 10.1097/00005373-199408000-00016 8064924

[B29] Foo GC Fractures of the zygomatic-malar complex: a retrospective analysis of 76 cases Singapore Dent J 1984 9 29 33 6599646

